# Evidence of Reactivity in the Membrane for the Unstable Monochloramine during MIMS Analysis

**DOI:** 10.3390/s18124252

**Published:** 2018-12-03

**Authors:** Essyllt Louarn, Abdoul. Monem Asri-Idlibi, Julien Leprovost, Michel Héninger, Hélène Mestdagh

**Affiliations:** 1 Laboratoire de Chimie Physique, UMR 8000, CNRS et Université Paris Sud, 91405 Orsay, France; idlibi.im@gmail.com (A.M.A.-I.); michel.heninger@u-psud.fr (M.H.); helene.mestdagh@u-psud.fr (H.M.); 2 AlyXan, Centre Hoche, 3 Avenue Condorcet, 91260 Juvisy-sur-Orge, France; julien.leprovost@alyxan.fr

**Keywords:** MIMS, monochloramine, FTICR, chemical ionization, in-membrane reaction, PTRMS, CIMS

## Abstract

Membrane Inlet Mass Spectrometry (MIMS) was used to analyze monochloramine solutions (NH_2_Cl) and ammonia solutions in a compact FTICR. Chemical ionization enables identification and quantification of the products present in the permeate. The responses of protonated monochloramine and ammonium increase linearly with the solution concentration. The enrichments were respectively 1.2 and 5.5. Pervaporation is dependent on pH and only the basic form of ammonia NH_3_ pervaporates through the membrane. Unexpectedly, the small ammonia molecule permeated very slowly. It could be due to interactions with water molecules inside the membrane that create clusters. Moreover, NH_2_Cl solutions, in addition to the NH_3_Cl^+^ signal, presented a strong NH_4_^+^ signal at *m*/*z* 18.034. Ammonia presence in the low-pressure zone before ionization is probable as NH_4_^+^ was detected with all the precursors used, particularly CF_3_^+^ and trimethylbenzene that presents a proton affinity higher than monochloramine. Ammonia may be formed inside the membrane due to the fact that NH_2_Cl is unstable and may react with the water present in the membrane. Those results highlight the need for caution when dealing with chloramines in MIMS and more generally with unstable molecules.

## 1. Introduction

Membrane Inlet Mass Spectrometry (MIMS) is an efficient tool to analyze water and air samples from the environment. It has gained interest since it was first reported in 1987 [1] as it allows real-time analysis of water and air samples by mass spectrometry [2] and generally preconcentration of targeted compounds. Its use spans from detection of organics in water to the more recent analysis of nitrites and nitrates [3]. MIMS technology is based on the separation of the analyte stream and the mass spectrometer inlet by a membrane. There are many possible configurations available: from in-vacuum membrane without any carrier gas to external tubular membranes using a dry carrier gas or a solvent, such as in condensed-phase MIMS [2]. Compared to more traditional GC-MS instruments used for volatile organic compounds (VOC) analysis, MIMS results in rapid analysis without complex preparation of the samples [4].

To make the MS easily portable, MIMS is usually associated to an electronic ionization (EI) source such as in underwater mass spectrometry [5,6]. The main drawback of the use of EI sources is the complexity of the mixture spectra as EI fragments each molecular ion into a large number of daughter ions, most of them being not specific of the molecule. On the contrary, chemical ionization (CI) methods produce low fragmentation, and then an easy-to-read mass spectrum without further separation [7,8]. In instruments such as Selective Ion Flow Tube (SIFT) [9] or Fourier Transform Ion Cyclotron Resonance (FT-ICR) [10], controlled conditions in the ionization zone enable direct quantification of the gas introduced. It is then possible to study the composition of the permeate gas behind the membrane, which gives access to a better understanding of the permeation process occurring during pervaporation and especially to enrichment factors and diffusion speeds.

This technique has been applied to the study of chloramine in water. Chloramination is an efficient way to decontaminate water before its use in public areas such as swimming pools or in refrigeration waters. Chloramine formation is processed by the mixing of an ammonia solution and sodium hypochlorite. Depending on the conditions (pH, temperature, Cl/N ratio, …), the solution contains monochloramine (NH_2_Cl), dichloramine (NHCl_2_) or trichloramine (NCl_3_) [11]. Monochloramine is formed for pH above 6.5. Its formation is optimal for pH 8 at 25 °C. Being highly reactive, monochloramine is the most efficient decontamination product.

Chloramine solutions are usually studied by spectroscopic or colorimetric methods [12]. However, as MS is a broadband technique and is able to analyse multiple compounds at the same time, there is interest for MS analysis of those compounds. Chloramines analysis by EI-MIMS was first presented by Kotiaho et al. in 1991 [13]. Subsequent studies using similar methods presented detection limits down to 0.08 mg/L [14,15] and down to 2 µg/L for a trap-and-release MIMS [16]. Senthilmohan et al. [17] presented in 2008 the analysis of monochloramine as a marker for inflammation by direct analysis of the exhaled breath gas at atmospheric pressure. Chemical ionization, alternatively by O_2_^+^, NO^+^ and H_3_O^+^, in a SIFT-MS instrument was used. The same group presented a heated system to improve signal a few years later [18].

We present here the study of ammonia and monochloramine by MIMS using CI and an FTICR instrument. In addition to the technical aspects of our development, there is much interest in observing and understanding the chemical interactions of the studied species within the membrane. Both monochloramine and ammonia presented unexpected behavior. This study addresses the reasons for those phenomena.

## 2. Results

### 2.1. Ammonia Permeation

#### 2.1.1. Diffusion and Enrichment

**Figure 1:**
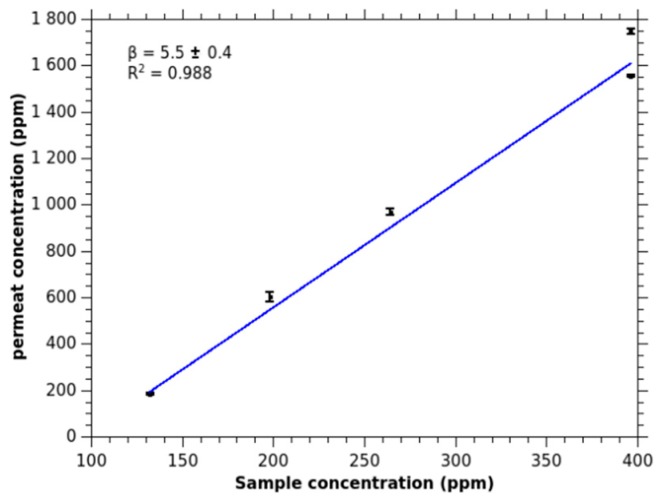
Calibration of ammonia signal as calculated from the ion signal (*m*/*z* 18.034) for different feed concentrations (molar fraction in ppm). The pH of the solutions are set to 10.5.

Ammonia is detected at a unique mass of 18.034 u corresponding to NH_4_^+^ ion. The temporal profile of NH_4_^+^ signal presents a regular step form, that is common in MIMS, denoting a limited uptake of the compound in the feed solution. Figure 1 presents the calibration curve of the signal for increasing ammonia concentrations for basic solutions. The response is linear and the enrichment β, defined as the slope of the curve, is 5.5 ± 0.3. As expected for a polar compound, the value is low. It is in the same order of magnitude as methanol [7,13].

The time response t_10–90%_, defined as the time to increase from 10% to 90% of the maximum signal, is nearly 20 min for the 125 µm PDMS membrane. The diffusion coefficient D_NH3_ of NH_3_ in PDMS at 20 °C is estimated from t_10–90%_ using Equation (1):

(1) $$t_{10–90\%}=0.237\frac{l^{2}}{D}$$

D_NH3_ is (0.027 ± 0.006) 10^−6^ cm^2^·s^−1^. This denotes a particularly slow diffusion rate, when compared to methanol which has approximately the same size as ammonia [7]: the diffusion coefficient of methanol under the same conditions is D_MeOH_ = 2.14 × 10^−6^ cm^2^·s^−1^. There is little data on ammonia diffusion in PDMS membranes: two different studies estimated the diffusion coefficient to 2.1 × 10^−6^ cm^2^·s^−1^ [19] and 4.2 × 10^−6^ cm^2^·s^−1^ [20] for pure gas samples. In the first study, the authors observed a considerably slower response when the gas in front of the PDMS was switched from ammonia to air, approximately three times slower than the ammonia increase when switching back to ammonia. They suggested that interactions between the membrane and ammonia molecules were in play.

The diffusion coefficient obtained in this study in aqueous solution is two orders of magnitude lower than for gas samples. The aqueous solution is probably the reason for that difference. The water being the solvent, there is no barrier to its permeation, the consequence is the swelling of the material due to the presence of water molecules in the free volume of the PDMS structure. Ammonia is known to form clusters in water. Formation of ammonia-water clusters [20,21] may explain the difference observed and the much longer permeation time determined in our experiments. Clusters of alcohols were described in PTFE polymers [22]. The main consequence of cluster formation was the long diffusion time in the polymer compared to non-clustering compounds such as acetone. In PDMS, methanol solutions do not react as methanol gas in PTFE, as it permeates rapidly and enrichment is low. It denotes a high diffusion coefficient and low solubility in PDMS [7]. The long time response obtained for ammonia in PDMS is not an expected feature for a small molecule, as it should behave like methanol. Clusters could form in the membrane or at the interface and diffuse slowly due to their large section. However, detection of clusters with our instrument is not possible as the low pressure maintained in the MS chambers is too low to keep the clusters structure and desolvation occurs before it reaches the ion source.

#### 2.1.2. pH Influence on Ammonia Signal

**Figure 2:**
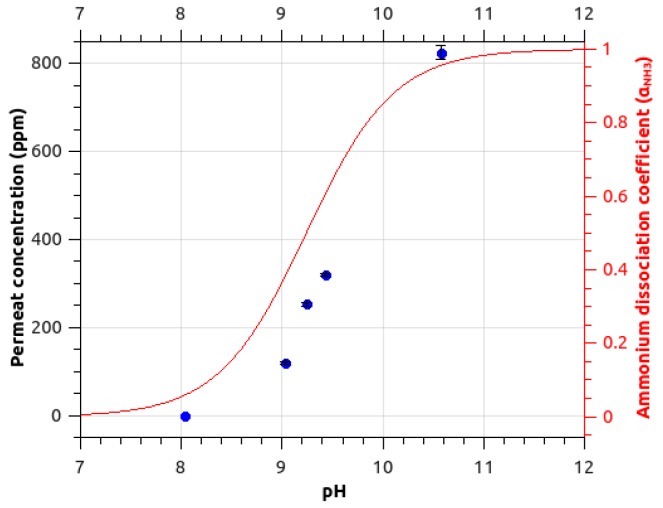
Ammonia signal (*m*/*z* 18.034) measured (blue points) for different pH around the pKa. The theoretical ratio of NH_3_ is superimposed as a red line (calculated for a pKa of 9.24) on the right scale.

Figure 2 presents evolution of ammonia signal for different pH values. The ammonia signal drops at acidic pH. The observed trend is similar to the variation of the ammonium dissociation coefficient α_NH3_ where:

(2) $$\alpha_{NH3}=\frac{K_{a}}{h+K_{a}}$$

where *K_a_* is the acidity constant (pKa = 9.24) and *h* is the hydronium ion concentration [H_3_O^+^]. The decrease of ammonia signal is centered on pKa. At a pH below the pKa, the ammonium form prevails in the feed solution. Ammonia is then in its ionic form NH_4_^+^, this prevents permeation of the species in the hydrophobic membrane. Ion formation in the feed solution inhibits the neutral species from transferring through the membrane. To enable ammonia detection in an aqueous solution, the pH has then to be set to values above the pKa.

### 2.2. Monochloramine Permeation

Protonated monochloramine presents two peaks at *m*/*z* 51.995 and *m*/*z* 53.992 due to the isotopic distribution of the chlorine atom. Signal of *m*/*z* 51.995 is higher than the *m*/*z* 53.992 signal as expected of the abundance of ^35^Cl versus ^37^Cl. At low concentrations, *m*/*z* 51.995 may be observed above the noise signal, whereas *m*/*z* 53.992 is not visible. Then, instead of considering the signal of protonated monochloramine NH_3_Cl^+^ as the sum of *m*/*z* 51.995 and *m*/*z* 53.992 signals, we preferred to reconstruct the signal from the major peak of the isotopic distribution, i.e., *m*/*z* 51.995. We then consider that:

(3) $$S\left[NH_{3}Cl^{+}\right]=\frac{S\left[m/z\;52\right]}{ab\left[{}^{35}Cl\right]}$$

where *S*[*NH*_3_*Cl*^+^] is the reconstructed signal of protonated monochloramine, *S*[*m*/*z* 52] is the signal of *m*/*z* 51.995 ion, and *ab*[^35^*Cl*] = 75.77% is the abundance of chlorine 35.

From the reconstructed signal, as we use chemical ionization, it is possible to evaluate the concentration of the permeate gas knowing the capture rate constant *k_C_* [18]. We used the value calculated by Senthiloman et al. [17]: *k* = 3.0 × 10^−9^ cm^3^·s^−1^.

**Figure 3:**
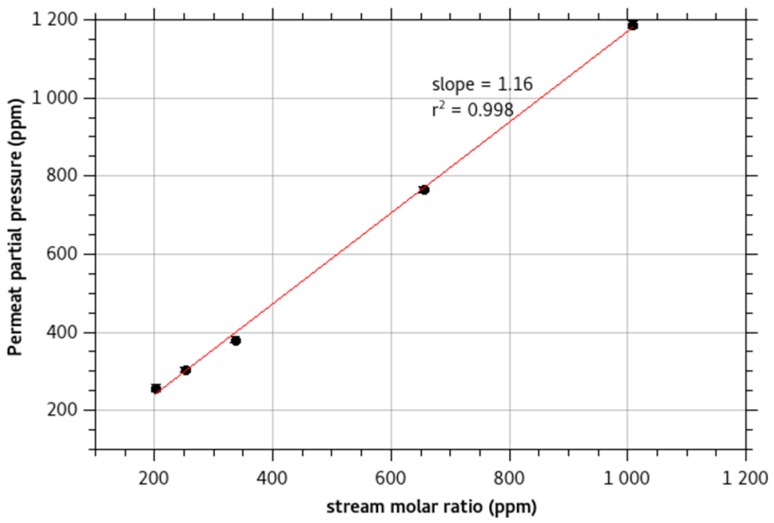
Monochloramine permeate concentration for different feed solution concentration. The line is the calculated calibration curve of the signal. The slope is the enrichment of the membrane.

Figure 3 presents the calibration curve of the concentration of monochloramine in the permeate gas for different monochloramine concentrations in the feed solution. The monochloramine signal presents a linear response. The enrichment β is the slope of the calibration curve. The value of the enrichment, β = 1.2, is low as monochloramine presents a moderate to slow diffusivity in PDMS and as it is soluble in water. It is in agreement with its low partition coefficient logKow estimated at −0.091 ± 0.350 (Calculated using Advanced Chemistry Development (ACD/Labs) Software V11.02).

**Figure 4:**
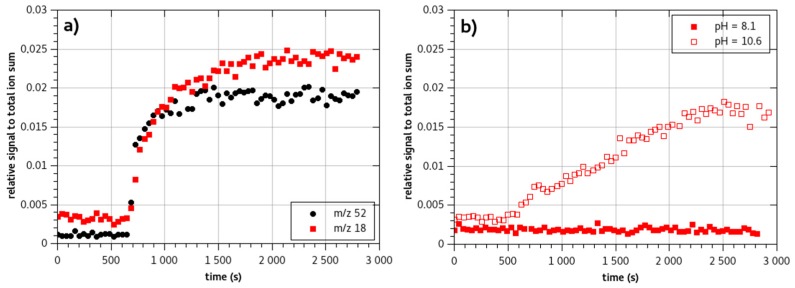
Time responses of the membrane system to (**a**) a solution of monochloramine (NH_3_Cl^+^ *m*/*z* 52 -black dots- and NH_4_^+^ *m*/*z* 18 -red squares- are presented) prepared at pH 8.1, (**b**) two ammonia solutions at pH 8.1 (filled red squares) and pH 10.6 (open red squares). Ammonia stock solution is added in the same quantity in the three solutions (solutions are prepared from 150 µL of ammonia stock solution in 200 mL water).

Monochloramine NH_2_Cl permeates through the membrane at a moderate rate. Contrary to steady state value, transient signal fitting is coarse as NH_2_Cl MIMS response is slightly different from a regular MIMS response. Indeed, first a fast response is observed, the *m*/*z* 51.995 signal increases rapidly. After that first phase, a second longer phase is observed before stabilization of the signal (Figure 4a). The presence of that second phase decreases the value of the diffusion coefficient.

## 3. Origin of the Observed NH_4_^+^ ion Signal for NH_2_Cl Solutions

Concomitantly to the appearance of the protonated monochloramine signal, in pure monochloramine solutions, a well-marked signal of NH_4_^+^ is measured. The values of the latter signal are as high as the NH_3_Cl^+^ signal (Figure 4a). Moreover, it presents a steady increase with increase of monochloramine concentration in the feed.

The presence of the NH_4_^+^ ion was not expected as earlier monochloramine MIMS experiments did not show this feature. Electron ionization (EI) is common in MIMS and monochloramine has been studied mainly using that ionization method. In EI-MIMS, NH_2_Cl^+^ was generally the only product formed at 70 eV. Moreover, the EI product NH_2_Cl^+^ is quite stable as CID measurement did not exhibit strong fragmentation [12]. NH_2_^+^ is formed but with a low branching ratio (7%).

The presence of that ion may be due to three different phenomena:

1. at the working pH, dissociation is partial and presence of *m*/*z* 18.034 is due to the transfer of ammonia in the PDMS membrane;
2. reactivity of the protonated monochloramine occurs in the ICR cell, either by dissociation or secondary reaction;
3. dissociation of the neutral monochloramine happens somehow in-between the solubilization step in the PDMS membrane and ionization in the ICR cell.

These hypotheses are successively studied below.

### 3.1. Is It Due to Permeation of NH_3_ from the Feed Solution?

As demonstrated previously (Figure 2), permeation of substances is dependent on the form of the species (neutral or ionic). The pH of the solution is then established to a value for which ammonia is represented by its acid form NH_4_^+^. The pH of the feed solutions were then all set to values below the pKa (pH 8.1). Using the same concentration of ammonia, we compared solutions of chloramine and ammonia at pH 8.1 (red squares on Figure 4a,b). No *m*/*z* 18.034 signal could be evidenced when analyzing the ammonia feed solution. On the contrary, the monochloramine solution exhibited a strong *m*/*z* 18.034 signal. The ammonia that may be present in the feed solution is then not bound to permeate through the membrane at the pH of the solution.

Another interpretation would be to consider that at the vicinity of the membrane, as monochloramine is consumed by solubilization in the PDMS, a disequilibrium would occur. The difference of solubilization should then modify the pH in the limit layer. The consumption of NH_2_Cl increases HO^−^ formation, pH may then increase locally as we didn’t use a buffer solution to set the pH. Figure 4 presents the time response of a chloramine solution (Figure 4a) and ammonia solution (Figure 4b) at the same concentration for a basic pH 10.6. The two solutions exhibit significantly different time responses for ammonia: the NH_4_^+^ signal from monochloramine analysis is at least three times shorter than the NH_4_^+^ signal from ammonia analysis. If ammonia were formed locally in the feed water solution, the time response of the *m*/*z* 18.034 from monochloramine solutions should be of the same order of magnitude or may be delayed compared to ammonia solution response. On the contrary, the NH_4_^+^ signal observed from monochloramine solution appears rapidly.

Therefore, it is probable that the NH_4_^+^ signal observed is not due to ammonia permeation itself through the membrane.

### 3.2. Is It Due to Ion-Molecule Reactivity?

When looking to MIMS response of NH_3_Cl^+^ and NH_4_^+^ for a monochloramine solution (Figure 4a), it appears that permeation times of both ions are quite similar. It is then possible that the observed NH_4_^+^ is due to a phenomenon occurring in the vacuum chamber after monochloramine pervaporation.

**Figure 5:**
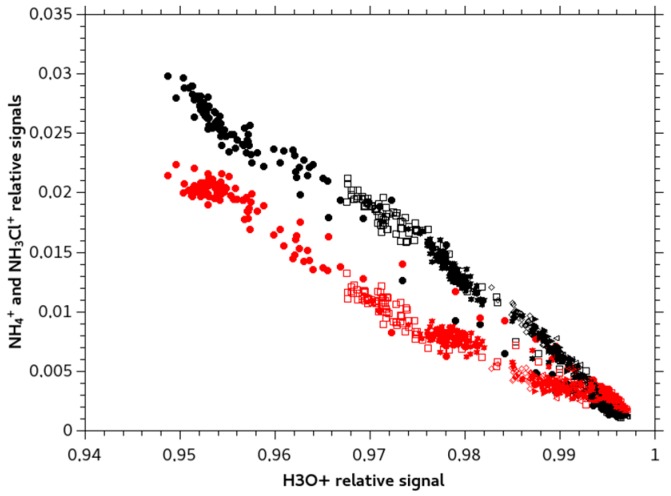
NH_4_^+^ (*m*/*z* 18) signal in red and NH_3_Cl^+^ (*m*/*z* 52) signal in black compared to H_3_O^+^ signal (precursor) during transient and stable MIMS response for different concentrations.

Figure 5 shows the dependency of the transient and stable response of the MIMS signal to monochloramine solutions of increasing concentrations with the H_3_O^+^ signal. The NH_4_^+^ signal presents a pattern consistent with a reaction scheme such as:

NH_2_Cl + H_3_O^+^ → NH_3_Cl^+^ + H_2_O (4)

→ NH_4_^+^ + HOCl (5)

NH_3_Cl^+^ + NH_2_Cl → NH_4_^+^ + NHCl_2_ (6)

All three reactions are exothermic [23,24]. Reactions (4) and (5) are parallel, as no delay for NH_4_^+^ formation was highlighted: at low reaction extent both ion products are observed. Reaction (6) is a secondary reaction that would explain the increase of NH_4_^+^ signal compared to NH_3_Cl^+^ signal for higher reaction extent.

As the permeate is mainly made of water, a reaction with water may be considered:

NH_3_Cl^+^ + H_2_O → NH_4_^+^ + HOCl (7)

From formation enthalpies [23,25], we calculated a reaction enthalpy of 0.5 kJ·mol^−1^. The reaction is thermoneutral, reaction rate coefficient is then likely to be slow. The yield of reaction (7) is then bound to be much lower than the amount of ammonia signal.

Unfortunately, the reactivity of NH_2_Cl and H_3_O^+^ is difficult to study as NH_2_Cl is nearly never formed as a pure product. To overcome this limitation, we proposed to study the products obtained using different precursors: 2 proton donor precursors (para-difluorobenzene F_2_C_6_H_4_ [26] and 1,3,5-trimethylbenzene (CH_3_)_3_C_6_H_3_) and a potential hydride or chloride abstraction precursor CF_3_^+^ [27]. Proton affinity (PA) of monochloramine is 797.05 kJ·mol^−1^ [23], it should then react by PTR with H_3_O^+^ as observed, and with F_2_C_6_H_5_^+^. On the contrary, PA of 1,3,5-trimethylbenzene is higher. The reactivity with that ion should then be limited. The reactivity of CF_3_^+^ is only known for NH_3_ [28]:

NH_3_ + CF_3_^+^ → CF_2_NH_2_^+^ + HF (8)

The kinetic rate coefficient is 2.1 × 10^−9^ cm^3^·s^−1^.

Interestingly, the formation of NH_4_^+^ is not prevented by a change of the precursor to higher PA (Table 1). Moreover, the observed products fit with a reactivity of ammonia with the PTR precursors, as the chosen precursors have all a PA below ammonia (AP(NH_3_) = 853.6 kJ·mol^−1^). Using CF_3_^+^, two products are also observed. The CF_2_NH_2_^+^ ion is very probably due to Reaction (9). Reaction of chloramine and CF_3_^+^ gives rise to a similar reaction:

NH_2_Cl + CF_3_^+^ → CF_2_NHCl^+^ + HF (9)

From those experiments, it appears that the ammonium signal is probably due to direct ionization of ammonia and not from Reaction (5).

Occurrence of Reaction (6) was also tested. First, NH_3_Cl^+^ ion is isolated in the ICR cell by the excitation and the ejection of the other ions present in the cell. Then, a pulse of permeate is injected in the ICR chamber. Time is left for the reaction to occur. Finally, no NH_4_^+^ was detected. Ricci and Rosi [23], studying the reactivity of NH_3_Cl^+^ produced by CI/CH_4_ in a FTICR apparatus, observed the ion NH_2_Cl_2_^+^ as a product of a secondary reaction (formed by Cl^+^ transfer). Likewise, they did not report NH_4_^+^ formation.

The observed ammonia signal is then probably not due to a secondary reaction of ionized NH_2_Cl. Those results tend to point to an alternate scenario: ammonia is present in the cell before ionization.

### 3.3. Is Neutral Monochloramine Reacting in the Vacuum Chamber?

From the previous experiments, we could not explain the presence of NH_4_^+^ peak from ammonia in the solution or from the ionization of monochloramine. Ammonia is then probably formed in-between from a neutral reaction:

NH_2_Cl + H_2_O ⇄ NH_3_ + HOCl (10)

The reactivity of neutrals on surfaces may take place inside the vacuum cell. However, probability of encountering a surface or a molecule is low in a low pressure FTICR. If such a phenomenon occurred, it would not explain the high proportion of *m*/*z* 18.034 peak. Moreover, one could argue that the problem is due to an inner problem of our instrument. That is partially true only. We tested our membrane system on two different FTICR instruments. Despite being similar, their internal structure and in particular the cell is different in terms of material used. Both exhibited a strong NH_4_^+^ signal proportional to NH_2_Cl concentration.

The probable reason for the presence of ammonia in the vacuum chamber is a reaction occurring in the membrane itself. As explained, ammonia is not permeating through the membrane as it is mainly under its ammonium form. This induces a disequilibrium inside the PDMS membrane with an excess of monochloramine compared to ammonia and hypochlorite. NH_2_Cl may then react with water inside the membrane to form NH_3_ and HOCl as in Equation (10). HOCl has a low proton affinity [30], it cannot react with the proton transfer precursors used in this study.

An in-membrane reactivity may explain why the ammonia signal appears at the same time as the monochloramine signal (Figure 4a). Besides, it appears that the *m*/*z* 18.034 signal generally keeps on steadily increasing even after monochloramine signal reached the steady state. This feature would be due to transfer of ammonia in the membrane from where it was formed originally. Diffusion of ammonia being slower than monochloramine, response of *m*/*z* 18.034 signal would involve two diffusion parameters, one due to monochloramine diffusion and the second due to ammonia diffusion in the membrane. The appearance of the ammonia signal is then a two-step process.

Finally, experiments of Provin and Fujii [31] in a microdevice suggested that reactivity inside a PDMS membrane between different species is possible with no chemical alteration of the membrane.

## 4. Materials and Methods

### 4.1. Chloramine and Ammonia Solutions

A stock solution of ammonia was prepared from 20 mL of concentrated NH_4_OH solution (28% Reagent grade, VWR Prolabo) adjusted to 50 mL with deionized water. To prepare ammonia solution for calibration, various volumes of the stock solution were added in a 200 mL vial, and pH was adjusted to 10.5.

A stock solution of sodium hypochlorite was prepared daily by diluting a purchased concentrated solution (14% active chlorine, VWR Prolabo) in deionized water. Sodium hypochlorite is known to be unstable in solution. Decomposition of HOCl occurs at moderate speed. Therefore, the sodium hypochlorite solution was titrated every week to evaluate its concentration.

Monochloramine was synthesized in-situ by addition of the diluted solution of sodium hypochlorite to the diluted ammonia solution at molar ratio 1:1 (ammonia/chlorine) under alkaline conditions (pH ~ 8.5). Different concentrations were achieved by preparing the solution using different volume of the two solutions and mixing them in a 200 mL vial completed with deionized water.

Under those conditions, only monochloramine should form through the reaction [14]:

NH_3_ + ClO^−^ ⇄ NH_2_Cl + HO^−^ (11)

During MIMS analysis, absence of di- or tri-chloramine was systematically checked.

Monochloramine is not stable in solution and produces NH_3_ or dichloramine in standard solutions. NH_2_Cl solutions were then prepared daily and the concentrations of monochloramine were measured in the solution using the DPD (*N*,*N*-diethylphenylene-1,4-diamine) method [32]. DPD reagent is oxidized by chloramine. The oxidation product is red and absorbs visible light at 510 nm. It is then titrated by spectroscopy or by ammonium and iron sulfate until total discoloration.

The pH of the solution was monitored by a Lab 860 pH-meter (Schott Instruments). Adjustment of pH value was obtained by addition of concentrated solutions of H_2_SO_4_ and NaOH.

### 4.2. Membrane Inlet

The membrane inlet has been described in a previous work [7]. In brief, the membrane material is made of a PDMS elastomer supplied by Goodfellow SARL (Lille, France). Different thicknesses are available. Optimal thickness is 125 µm as thinner membranes break easily under our experimental conditions. The PDMS film is held between two stainless steel plates. A channel 40 mm × 1.5 mm channel is extruded in each plate. The interface between the sample stream and the vacuum cell is then 60 mm^2^. Two hollow copper sheets, filled with water at 20 °C from a controlled temperature water bath, set the temperature of the membrane system.

One side of the membrane is connected to the water feed at 80 mL·min^−1^, and the other side is directly connected to the vacuum chamber of the mass spectrometer.

### 4.3. Compact Low-Field FT-ICR Mass Spectrometer and Chemical Ionization

The mass spectrometer is based on a Fourier transform ion cyclotron resonance (FT-ICR) analyzer. The coupling and performance of the instrument have been extensively described elsewhere [7,26,27,33,34]. The instrument has two vacuum chambers: the ICR vacuum chamber and the waste chamber. The latter enables generation of a continuous flow at low pressure. The two vacuum chambers are linked to the MIMS permeate exit by a three-way valve that directs the flow to the buffer vacuum chamber, for continuous pumping of the membrane, or to the ICR vacuum chamber, for a few 100 ms during ionization.

This technology enables chemical ionization in the ICR cell. Ion formation occurs by means of sequential operations in 3 to 5 s. Briefly, it is as following: 1. neutral precursor is introduced: H_2_O for example; 2. molecules are ionized by an electron beam at 70 eV: H_2_O^+^ is formed; 3. time is left for secondary reactivity to occur: ion-molecule reactivity of H_2_O^+^ and H_2_O produces H_3_O^+^; 4. permeate is introduced for a few 100 ms: permeate contains a molecule M; 5. time for reactivity to occur: proton transfer reaction gives MH^+^; 6. detection.

For this study different precursors were used. H_3_O^+^ is formed from H_2_O directly by electron ionization [10]. F_2_C_6_H_5_^+^ and (CH_3_)_3_C_6_H_4_^+^ are formed by proton transfer reaction of the neutral, respectively 1,4-difluorobenzene and 1,3,5-trimethylbenzene, and H_3_O^+^, adding a repetition of step 4 and 5, one for the precursor formation (M is the precursor) and one for permeate introduction [26]. CF_3_^+^ is formed by electron ionization of CF_4_ [27].

Two compact FTICR mass spectrometers have been used: Btrap2, for the study on different precursors, and MICRA (mobile ICR analyzer) [18] for the other experiments. Btrap2 is an instrument developed by AlyXan (Juvisy-sur-Orge, France): it is fully automated and uses a 1 T permanent magnet. MICRA’s magnet is 1.24 T.

The use of an FTICR makes it possible to measure exact masses, with an accuracy of 0.01 u for Btrap2 and 0.005 u for MICRA, and to identify the ions. Typically, we are able to differentiate NH_4_^+^ (18.034 u) and H_2_O^+^ (18.011 u). Quantification of the compounds in the permeate is possible since ion-molecule reaction conditions are controlled in the cell (pressure, temperature, reaction time) [10]. Rate coefficients for ammonia [35] and monochloramine [17] are taken from literature.

## 5. Conclusions

This study presents new results on chloramine and ammonia detection by MIMS in aqueous solutions. The steady state response of ammonia and monochloramine are in accordance with their high solubility in water. However, the products detected for monochloramine sample and the time responses for both samples were not as expected for low molecular mass species.

First, a long time response was observed for ammonia solutions. Presence of water in the membrane is probably the reason for that result, as it induces the formation of ammonia clusters, what would drastically decrease the diffusion rate by increasing the ammonia cross section.

Second, in PTR mode, monochloramine feed solution presented two ion products: protonated monochloramine NH_3_Cl^+^ and NH_4_^+^. Whereas the first ion is the common protonated MH^+^ ion, the latter ion NH_4_^+^ was not expected. The *m*/*z* 18.034 ion signal appeared at the same rate as the NH_3_Cl^+^ signal and its intensity was in the same order of magnitude as NH_3_Cl^+^ signal. As the pH was below pKa, the NH_4_^+^ was not due to ammonia permeation through the membrane. We studied the reactivity of the permeate gas and neutral monochloramine with various precursors. All exhibited a signal due to ammonia reactivity. Reactivity of the neutral NH_2_Cl may then occur, probably in the membrane. NH_2_Cl is an unstable compound that tends to dissociate in an excess of water into NH_3_ and HOCl. This phenomenon may take place in the membrane as the medium lacks of ammonia.

The presence of the protonated ammonium ion has to be acknowledged when measuring monochloramine in real samples. pH of those solutions are not all set to a specific value, particularly when working in real-time, ammonia may then be present under its basic form. Moreover, presence of ammonia from different sources in the solutions is probable. The feature presented here leads to take with caution the precision of the measurement for such solutions as the ratio of NH_3_Cl^+^ and NH_4_^+^ is not steady and vary slightly with concentration. Direct sampling of headspace may be a better method, as stability of monochloramine in air is probably higher and as preconcentration effect of the MIMS system for monochloramine is limited.

More generally, the membrane in MIMS has to be taken into account when interpreting the data obtained. It should not be considered as a black box that has no influence on the products obtained. The membrane is a chemical matrix that could be involved in the reactivity of the studied compounds as observed in our study.

## Figures and Tables

**Table 1:** Products observed by ion-molecule reaction of the permeate of a monochloramine solution and 4 precursors. 3 of them are proton transfer precursors.

Neutral Precursor	Ionic Precursor	PA [29] (kJ·mol^−1^)	Product 1	Product 2
H_2_O	H_3_O^+^	691	NH_3_Cl^+^	NH_4_^+^
F_2_C_6_H_4_	F_2_C_6_H_5_^+^	718.7	NH_3_Cl^+^	NH_4_^+^
(CH_3_)_3_C_6_H_3_	(CH_3_)_3_C_6_H_4_^+^	853.6	-	NH_4_^+^
CF_4_	CF_3_^+^	-	CF_2_NHCl^+^	CF_2_NH_2_^+^

## Abbreviations

MIMS	Membrane Inlet Mass Spectrometry
PDMS	Polydimethylsiloxane
VOC	Volatile Organic Compound
EI	Electronic Ionization
CI	Chemical Ionization
FT-ICR	Fourier Transform Ion Cyclotron Resonance
SIFT	Selected Ion Flow Tube
PTFE	PolyTetraFluoroEthylene
PTR	Proton Transfer Reaction
